# Fatty Acids and Calcium Regulation in Prostate Cancer

**DOI:** 10.3390/nu10060788

**Published:** 2018-06-19

**Authors:** Ivan V. Maly, Wilma A. Hofmann

**Affiliations:** Department of Physiology and Biophysics, Jacobs School of Medicine and Biomedical Sciences, University at Buffalo, 955 Main Street, Buffalo, NY 14203, USA; ivanmaly@buffalo.edu

**Keywords:** androgen independence, bone, castration resistance, exosomes, hypoxia, metastasis, myosin IC, obesity

## Abstract

Prostate cancer is a widespread malignancy characterized by a comparative ease of primary diagnosis and difficulty in choosing the individualized course of treatment. Management of prostate cancer would benefit from a clearer understanding of the molecular mechanisms behind the transition to the lethal, late-stage forms of the disease, which could potentially yield new biomarkers for differential prognosis and treatment prioritization in addition to possible new therapeutic targets. Epidemiological research has uncovered a significant correlation of prostate cancer incidence and progression with the intake (and often co-intake) of fatty acids and calcium. Additionally, there is evidence of the impact of these nutrients on intracellular signaling, including the mechanisms mediated by the calcium ion as a second messenger. The present review surveys the recent literature on the molecular mechanisms associated with the critical steps in the prostate cancer progression, with special attention paid to the regulation of these processes by fatty acids and calcium homeostasis. Testable hypotheses are put forward that integrate some of the recent results in a more unified picture of these phenomena at the interface of cell signaling and metabolism.

## 1. Introduction

With over 3 million men living with the condition in the United States alone, prostate cancer is one of the most common malignancies that afflict the male population [1,2]. While the numbers reflect this cancer’s comparative amenability to early diagnosis and its long period of relative indolence in many of the patients, the disease is at the same time characterized by a transition to the disseminated and lethal stages for which the prognosis and treatment remain difficult, despite considerable progress achieved in the molecular and clinical science in recent years [3]. Specifically, the emergence of castration resistance and metastasis separate the difficult-to-manage stages from the disease that is believed to merit only watchful waiting [4], and during these late stages, quality of life is limited in large measure by the complications connected with metastasis to the bone.

Molecular biological research has the potential to identify the markers that are prognostic of the disease progression and could guide individualized treatment and treatment prioritization among the patients with this heterogeneous condition. At the same time, epidemiological research has established a correlation of not only the disease incidence, but also its progression with nutrition, particularly with the intake of fatty acids and calcium. The present review builds on the latest conceptualizations of the role of dietary-fat regulation [5] and fatty acid metabolism [6] in prostate cancer. It also extends the recent systematization of the intracellular calcium signaling pathways in prostate cancer [7] by adopting a tissue-level perspective on the main checkpoints in prostate cancer progression—castration resistance, vascularization, and bone metastasis.

## 2. Dietary Intake and Intracellular Signal Conversion

### 2.1. Co-Consumption of Fatty Acids and Calcium

Considerable effort has been directed in recent years toward characterization of the regional differences in the occurrence of prostate cancer and disease progression, and their possible connections with the local diet. The Japanese case, the Mediterranean diet, and the South American pattern of meat consumption have been given special attention, as they relate to fatty acids and calcium. In Japanese men [8], a prospective study showed that intake of saturated fatty acids and calcium via dairy products was associated with the development of prostate cancer. The correlation was especially significant for palmitic and myristic acid. These results appeared to supersede the earlier and broader retrospective analysis that was unable to show any association with dairy intake [9]. The cohorts followed in these studies are informative because of the low incidence of prostate cancer in Japan and low consumption of dairy products. Most recent work, however, shows that the uncovered pattern is not limited to this region. Post-diagnosis consumption of whole milk, especially in overweight men, was found to associate with the risk of prostate cancer recurrence and disease-specific mortality in American patient cohorts [10,11]. Similar results have been obtained on a patient population from Sweden, where milk consumption is high, yet high-fat milk intake still correlates with prostate cancer progression [12].

Adherence to the Mediterranean diet is associated inversely with prostate cancer incidence [13]. This traditional diet is thought to be characterized by a moderate consumption of milk products dominated by low-fat dairy [14]. While the total fat content approaches that of the modern Western diet (~30% of the caloric intake), the proportion of saturated fat is low (8%) [15]. The positive effect of the Mediterranean diet in the cited recent study was qualitatively attributable, in part, to the high content of *n*-3 fatty acids from fish, while the dairy and red-meat components with saturated fatty acids were seen as contributing unfavorably. Earlier work, however, showed that the outcome post-diagnosis (metastasis and prostate cancer-related mortality) is not associated with the Mediterranean diet adherence [16]. Interestingly, some of the discrepancies among the studies that reported both presence and absence of prostate cancer risks association with calcium intake [17] could potentially be related to the fact that risk is associated with calcium intake from dairy products but not from non-dairy sources [18].

Similarly to the red-meat component of the Mediterranean diet, the traditional Argentinian diet, which is distinguished by high red-meat content, has been found to be associated, as compared with alternative diets in the same population, with the risk of prostate cancer [19]. These results appear to supersede the earlier and collectively less conclusive work on South American diets [20]. The traditional diet of the Southern Cone region is interesting, due to red-meat consumption reaching 50% of the calorie intake [21]. The cited recent study shows that the traditional pattern of the Argentinian diet correlates strongly with calcium intake. At the same time, one of the alternative local dietary patterns, termed the cheese pattern, was correlated not only with calcium, but also with conjugated linoleic acid. The latter was deemed to have protective qualities [22,23], and the cheese pattern overall was not correlated with prostate cancer [19]. Meat in the Southern Cone diet is also a major source of fat [21], contributing primarily saturated fatty acids and linoleic acid, while the main dietary oil consumed in this population is sunflower oil comprising ω-6 (linoleic) and ω-9 (oleic) fatty acids. In sum, among the most characteristic, widespread diets of the world that have been studied in relation to prostate cancer, there is evidence for co-consumption of calcium and fatty acids and its correlation with the disease incidence.

### 2.2. Conversion to Intracellular Calcium Signaling

The dietary-calcium effect on intracellular signaling, mediated by the calcium-sensing receptor (CaSR), is a comparatively well-researched area that will be reviewed in detail in connection with the mechanisms of bone metastasis (Section 6). In the light of recent work in prostate cancer and other fields, it also appears likely that free fatty acids may induce or modulate the calcium signaling in epithelial prostate cancer cells via the scavenger receptor CD36. The connection of this receptor to prostate cancer mechanisms was first established in studies of tumor vascularization. CD36 has been identified as the surface receptor in endothelial cells that mediated the apoptotic response to the angiogenesis inhibitor thrombospondin-1 [24]. Remarkably, however, both the blood vessels and the tumor cells in metastatic human prostatic carcinomas express CD36, as detected by immunohistochemistry [25]. In vitro, this receptor mediates the stimulatory effect of thrombospondin-1 on the invasivity of epithelial prostate cancer cells [26]. In this connection, it is intriguing that CD36 mediates epithelial-mesenchymal transition (EMT) in hepatocellular carcinoma cells under the action of specific free fatty acids, which bind and are taken up via this receptor [27]. Below, we lay out the evidence that suggests the likely existence of a similar fatty-acid-sensing mechanism in epithelial prostate cancer cells and the involvement of intracellular calcium as a second messenger.

Among the signaling pathways that have been identified downstream of CD36 in various cell types, the calcium signaling pathway characterized in the context of the murine gustatory perception [28] appears especially relevant, because it involves the ORAI1/3 heterodimer that determines the oncogenic switch in prostate cancer [29]. Similarly to the prostate cancer situation, the heterodimer in the gustatory papillae is gated by arachidonic acid and leads to store-operated calcium influx. Arachidonic acid in the taste bud cells is produced downstream of the linoleic acid binding to CD36 and phospholipase A activation. The pathway implicating CD36 in the arachidonic acid liberation is by no means unique to the taste bud cells, as it has been demonstrated in cell types as different as ovary epithelial cells and macrophages [30]. It seems likely that it may be operative in prostate cancer as well, providing for the modulation of intracellular calcium signaling by the fatty acid receptor on the plasma membrane. Detection of this mechanism of direct cross-talk between fatty acids and calcium homeostasis in the prostate epithelium or metastases and characterization of its possible role in the prostate cancer progression appear to be promising directions for further research.

## 3. Mechanisms of Castration Resistance

### 3.1. First-Line Therapy and the Escape Mechanisms

Ligand-dependent and other molecular interactions of androgen receptor (AR) play a pivotal role in the development of prostate cancer and its progression to lethal forms. AR is a nuclear hormone receptor whose most abundant ligand is testosterone [31,32]. In the prostate gland, testosterone is converted into an even more potent AR ligand, dihydrotestosterone (DHT) [33]. Minor androgens are also synthesized in the adrenal gland [34]. Overexpression of AR’s wild-type form in mouse prostate epithelium (under the probasin promoter) is sufficient to cause the type of neoplastic abnormality that is associated with early development of prostate cancer—prostate intraepithelial neoplasia (PIN) [35]. In the treatment of human prostate cancer, androgen ablation is one of the first-line therapies. It is achieved by orchiectomy, administration of gonadotropin-releasing hormone analogs, or both [36]. The disease’s escape from under the control of this therapy is, for many patients, the main checkpoint on the path to metastasis and unfavorable outcome [4]. The tumor’s acquired ability to grow under the conditions of reduced androgen availability is referred to castration resistance.

For the study of the molecular mechanisms behind the androgen deprivation effectiveness, it is of interest that isolated prostate epithelial cells placed in these conditions merely stop proliferating [37,38]. At the same time, tissue reconstitution experiments suggest that androgen-deprived stroma of the prostate gland can induce apoptosis in the epithelium [38]. Data obtained using the transgenic adenocarcinoma of the mouse prostate (TRAMP) model support this mechanism. Conditional deletion of AR in both the epithelium and stroma in TRAMP mice results in smaller tumors than the epithelium-targeted deletion alone [39]. Interestingly, the cellular mechanisms of tumor suppression may be to some extent species-dependent, as the histological analysis of human androgen deprivation material indicated a prevalence of cellular injury that was distinct from the signs of apoptosis [40].

The molecular mechanisms underlying the eventual acceleration of tumor growth, which is seen in many patients despite the continuing androgen ablation, appear to be numerous and, most likely, to vary between the individual cases. Central to the emergence of castration resistance is modulation of the AR activity that alters its dependence on ligand-binding. So, tyrosine phosphorylation enhances AR transcriptional activity [41], while MAPK signaling activation [38] and a *Pten* knock-out [42]—the experimental conditions that mimic the common molecular perturbations in prostate cancer [7]—can induce androgen independence in tissue reconstitution assays in an AR-dependent manner. The effect is so strong that *Nkx3.1*; *Pten* double mutants even exhibit castration resistance of PIN. The AR transcriptional activity is also positively regulated by ubiquitination via the E3 ligase ring finger 6 (RNF6), which is overexpressed in hormone-refractory human prostate cancer tissue [43]. An interesting link exists also between the emergence of castration resistance and the inflammatory response in the tumor microenvironment. Interleukin-1β produced by macrophages triggers depression of the AR corepressor complex, inverting the AR response to antagonists [44]. Inflammatory B-cell interleukins can also trigger both castration resistance and metastasis via translocation of IKKα to the nucleus [45]. As reviewed in detail below, these processes exhibit dependence on calcium and regulation by fatty acids.

### 3.2. Calcium Regulation

Several aspects of the intracellular calcium regulation have been uncovered in recent studies of AR signaling in prostate cancer. Notably, the calcium-binding protein calmodulin protects AR from degradation by the calcium-activated protease calpain [46,47,48]. This action involves direct binding of calmodulin to AR and the two proteins’ colocalization in the nucleus. The nuclear import of AR is normally induced by androgen binding [49]. Immunohistochemistry of xenografts transitioning to androgen-independent growth, however, revealed that the loss of nuclear localization upon androgen-deprivation was followed by its restoration, as the tumor acquired resistance characteristics [50]. These findings suggest the possibility that the calmodulin-dependent mechanism may add to the restoration of AR functionality following the androgen-deprivation treatment and emergence of castration resistance. At the same time, the outcome of the interplay of the calcium-dependent degradation and protection mechanisms may be disease context-specific and individual, and present interest for future quantitative research. According to recent work [51], the AR stability regulation by calmodulin is complemented by the latter protein’s ability to regulate phosphorylation of the N-terminal domain of AR, leading to suppression of this factor’s binding to the regulatory elements. Promisingly, pharmacological targeting of this additional function of calmodulin reversed some of the gene expression signature associated with prostate cancer [51].

In addition to its roles in WNT and Notch signaling pathways regulating the progression of prostate cancer (most recently reviewed [7]), calcium-calmodulin kinase (CAMK) II in prostate cancer cells was shown to mediate the activation of protein kinase AKT under the conditions of reduced AR expression [52]. CAMKII, in turn, is under negative regulation from AR (especially the kinase’s β and γ isoforms), and its overexpression promotes cell growth under androgen-free conditions. Taken together, these results underscore a pivotal role of CAMK as an integrator of calcium signals linked to the nuclear functions in prostate cancer.

Besides calmodulin, other calcium-binding proteins are known to play a role in the prostate cancer progression. The EF-hand calcium-binding protein S100P [53] was identified as the most overexpressed protein in xenograft models among hormone-refractory vs. primary untreated strains of CWR22 prostate cancer cells [54]. Conversely, manipulation of its expression levels in vitro and in xenograft models impacts growth and resistance to apoptosis induced by the chemotherapeutic topoisomerase inhibitor camptothecin [55]. The overexpression of S100P, in particular, was marked by an upregulation of MAPK14, epidermal growth factor (EGF) receptor (EGFR), WNT7B, matrix metalloproteases (MMP), and AR. S100P expression itself was shown to be androgen-dependent in androgen-responsive cells in vitro [56]. Upon the emergence of androgen independence in xenograft models, however, S100P was found to be re-expressed in the tumors [57]. It should be mentioned that there has been a conflicting report showing a degree of negative correlation with prostate cancer progression on human samples obtained prior to any hormonal treatment [58], and that S100P has been identified as a differential diagnostic marker of urothelial carcinomas of the bladder, concerning cases where the tumor mass is difficult to differentiate from a similarly advanced prostate cancer [59]. This calcium-binding protein has also been implicated in the colon [60] and pancreatic cancer. In the latter setting, monoclonal antibodies that block its in-vitro activities have been successfully tested in subcutaneous and orthotopic models [61]. The protein therefore may be a viable therapeutic target in prostate cancer as well.

The most recent proteomic analyses uncovered an enrichment of extracellular vesicles shed by androgen-deprived prostate cancer cells in vitro with plasma membrane calcium-transporting ATPase 1 [62]. This finding is intriguing in the context of the recently begun characterization of the functional consequences of protein and miRNA transfer mediated by extracellular vesicles in the progression to the migratory phenotype [63] and in the emergence of drug resistance [64,65] in prostate cancer. In connection with androgen deprivation specifically, it was determined earlier that exposure to CD9-enriched vesicles that had been secreted by the DHT-stimulated cells enhanced proliferation of the androgen-deprived cell culture [66], while the vesicles themselves were judged to be free of androgen. In this context, the finding of a calcium pump enrichment in the vesicles suggests that, potentially, calcium homeostasis too may be modulated by the exchange of extracellular vesicles-associated proteins between subpopulations of cells within the androgen-deprived tumor microenvironment.

### 3.3. The Feedback Model

Analysis of the mouse and in-vitro models [67] has suggested an establishment of positive feedback during the development of castration resistance. In this feedback loop, AR signaling upregulates and drives to CAMK kinase 2 (CAMKK2), while CAMKK2 potentiates AR transcriptional activity. The AR-dependent gene products, whose expression was linked to the feedback loop, included prostate-specific antigen (PSA) and the cell cycle proteins cyclin D1 and Rb. This finding is in agreement with the microarray data that revealed an elevated expression of CAMKK2/β in prostate cancer and earlier in-vitro results that demonstrated an induction of the kinase’s expression by androgen [68]. The data also supported the existence of a cascade downstream of AR that involves CAMKK and AMP-activated protein kinase, and showed that this cascade mediates the androgen stimulation of the cells’ migration and invasion [68], as well as their central metabolism [69]. Further supporting the feedback model, immunohistochemistry demonstrates that a return of CAMKK2 expression accompanies the emergence of the castration-resistant disease [69].

### 3.4. Bidirectional Connection with Fatty Acid Metabolism

**Figure 1:**
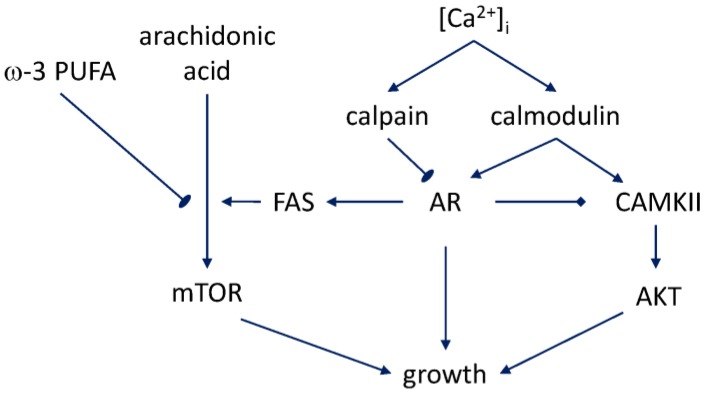
Some of the pathways involved in the emergence of androgen-independent growth.

Recent work has begun to clarify the mechanisms of bidirectional regulation that connect androgen signaling with fatty acid metabolism (Figure 1). The basis for this interaction network was identified in the study of the expression and activity of fatty acid synthase (FAS), the enzyme that is upregulated in prostate cancer and associated with disease grade [70]. In androgen-responsive prostate cancer cells, including those with aberrant androgen sensitivity, androgen stimulation was found to lead to a significant upregulation of FAS on the mRNA level and an even larger increase of the enzyme’s activity [71]. The FAS upregulation precedes the characteristic accumulation of lipid droplets in the prostate cancer cells [72]. Lipid droplets are an important functional marker because of their involvement in fatty acid-regulated cholesterol metabolism, which, in turn, impinges on AR signaling. As sites of cholesterol ester storage, in vivo they demarcate advanced prostate cancer, and in vitro, the cholesterol ester-rich androgen-independent and high-passage prostate cancer cells [73]. Such cells, the same recent work has shown, no longer exhibit the key step in androgen signaling—nuclear translocation of AR. The dominant species in the lipid droplets is cholesteryl oleate. However, depletion of cholesterol ester in these experiments caused an inhibition of uptake of arachidonic acid as well, and was causative of the diminished aggressiveness that was seen in xenografts. The mechanism leading to the cholesterol ester accumulation downstream of the PTEN loss, which is characteristic of prostate cancer cells, involves, according to this study, the PI3K/AKT/mTOR pathway. This finding dovetails with the earlier result that ω-3 fatty acids counteract the effect of arachidonic acid and inhibit progression to hormone independence via suppression of mTOR signaling [74].

The branched and redundant character of the fatty acid–AR interaction network is demonstrated by the fact that the cholesterol ester accumulation is linked to increased low density lipoprotein (LDL) accumulation via LDL receptor (LDLR) [73], and not exclusively to FAS upregulation. In connection with the most recently investigated role of exosome secretion in the lateral transfer of the aggressive phenotype among prostate tumor cells [64,75,76,77,78], the LDLR-mediated pathway appears to be of special interest. The entry of LDL via LDLR is followed by its traffic to the late-endosome compartment on the way to hydrolysis into free fatty acids and cholesterol and the cholesterol ester synthesis [79]. It is conceivable, therefore, that the fatty acid uptake perturbation caused by the cholesterol ester accumulation at the late stages of prostate cancer progression may alter the fatty acid composition also of the late-endosomal compartment and, consequently, the exosomal membranes that originate there [80]. This possibility is suggestive when juxtaposed with the most recently characterized mechanism of exosome secretion in prostate cancer cells, which implicated the aggressive prostate cancer-specific isoform A of myosin IC in the secretion of MMP-containing exosomes [81]. The effectiveness of secretion was found to be dependent on the lipid-binding domain of the molecular motor, and it is likely that the exact fatty acid composition of the vesicular membrane may have a quantitative effect as well. The role of this composition in the invasive potential of prostate cancer and in the changes accompanying the establishment of castration resistance is a promising avenue for future research.

## 4. Resistance to Growth Inhibition

Among the aspects of signaling in which calcium impinges critically on the physiological hallmarks of tumor cells [82,83], the emergent insensitivity to growth inhibitors plays a pivotal role. Transforming growth factor (TGF) β (TGF-β) is a growth inhibitor for many epithelial cells that becomes a tumor promoter in advanced cancers through alterations of the associated signaling [84]. In the prostate, this path is followed via a transformation of the TGF-β signaling—a proliferation inhibitor and apoptosis inducer in the normal epithelium—into a tumor-promoting mechanism that acts extraepithelially through neoangiogenesis, extracellular matrix remodeling, and immunosuppression [85]. Its suppression and proapoptotic action on the cancerous epithelium appears to be reduced by means of downregulation of the receptor expression. Expression of both TGF-β receptor type I and II in epithelial prostate cells correlated inversely with the tumor grade in immunohistochemical studies [86]. Similar results were obtained on the mRNA level [87]. Further supporting this paradigm, expression of the dominant-negative TGF-β receptor in the SV40 large T antigen transgenic mouse model of prostate cancer causes increased metastasis [88]. Although Smad2 has been shown to have tumor suppressor potential, with its mutations impacting the apoptosis signaling and inducing tumorigenicity in a rat prostate cell line [89], analysis of this downstream TGF-β intermediary’s expression and potential mutations in human prostate cancer samples showed no abnormality [90].

In culture, prostate carcinoma cells (PC-3U) retain the ability to respond to a stimulation with TGF-β with signs of an apoptosis initiation, displaying both a calcium transient and mitochondria depolarization [91]. This finding of calcium-transient induction is consistent with TGF-β effects on other cell types, such as hepatocytes [92] and renal mesangial cells [93], as well as on primary pulmonary fibroblasts [94], which exhibited calcium waves, and on osteoblasts [95], which display oscillations. In other epithelial cells, the evidence for the involvement of calcium in inhibitory TGF-β signaling comes from experiments on keratinocytes, in which nucleolin-mediated nuclear translocation of the S100C/A11 calcium-binding protein is a shared feature of the response to calcium elevation and TGF-β [96,97,98]. An intriguing interaction during the metastatic progression with altered TGF-β signaling in the tissue context, however, is suggested by MMP9 acting as an activator of the latent TGF-β complex, as was shown on murine mammary epithelial cells and on purified proteins in vitro [99]. MMP9 plays a significant role in prostate cancer progression [100] and is secreted in association with the motor protein myosin IC [81], whose nucleocytoplasmic distribution in prostate cancer cells is regulated by calcium [101]. Consistent with this connection to the extracellular matrix remodeling, TGF-β is associated in prostate cancer patient samples with metastasis, angiogenesis, and poor prognosis [102]. Comparison of benign and malignant (e.g., RWPE1 and PC3) prostate epithelial cell cultures showed that the latter are capable of autoinduction of TGF-β production via extracellular signal-regulated kinase (ERK) activation due to a defective recruitment of phosphatase PP2 to TGF-β receptors [103]. It is possible that the calcium transient at the same time limits secretion of metalloproteases by sequestering myosin IC in the nucleus. The primary effect may be rendering epithelial cells insensitive to the locally produced TGF-β while permitting its activation and action in the stroma. Additionally, under the hypothesis put forward in the last section, the process may be under kinetic control by the fatty acid content of the secretory membranes.

AR physically interacts with Smad3 and, in a complex with DHT, prevents association with Smad-binding elements [104]. The crosstalk is bidirectional, with Smads affecting AR transcriptional activity [105] variously, depending on the cellular and molecular context [106]. The androgen-bound AR, however, specifically suppresses the expression of TGF-β receptor II [107]. Androgen deprivation therapy in the mouse model with a dominant-negative TGF-β receptor [108] led to an increased nuclear localization of AR, pointing to the role of the abnormal TGF-β signaling in the emergence of castration resistance. At the same time, the downstream TFG-β effectors β-catenin and Smad4 were also found to translocate to the nucleus in an enhanced fashion. The latter effect was linked to an induction of transcription factors involved in EMT. Running contrary to the overall theme of progression, however, the retained ability of metastatic cell lines to respond to TGF-β as an inhibitor of growth (as well as of motility and invasion) has been demonstrated by experiments on LNCaP and the castration-resistant C4-2 and C4-2B lines [109], which all displayed the TGF-β1-induced phosphorylation and nuclear translocation of Smad2/3. These aspects of the calcium-linked TGF-β signaling in prostate cancer cells deserve further investigation, which may potentially uncover how the above hypothesized connections to the fatty acid and unconventional myosin regulation could “tip the scales” in the pro- and anti-growth signal transduction.

## 5. Angiogenesis and Hypoxia

### 5.1. Calcium-Mediated and Fatty Acid-Regulated Hypoxia Responses

Sustained angiogenesis is another recognized biological hallmark of cancer that makes tumor growth possible. Development of regions of low oxygen availability is characteristic of solid tumors, including prostate cancer, as is the expression of elevated levels of hypoxia-induced transcription factors—HIFs [110,111]. As was shown originally on a hepatocellular carcinoma cell line [112], calcium, in an ionomycin-inducible and 1,2-bis(*o*-aminophenoxy)ethane-*N*,*N*,*N*′,*N*′-tetraacetic acid (BAPTA)-sensitive fashion, potentiates secretion of vascular endothelial growth factor (VEGF) and transcriptional activity of the primary hypoxia response regulator, HIF1α, without changing the factor’s own expression level. This effect is sensitive to calmodulin inhibition, whether through a dominant-negative mutant or the use of the inhibitor (W7), and depends on pERK. Any future calcium-linked therapeutic approach will have to take into account the fact that the proliferative response to VEGF is itself calcium-mediated, via the phosphorylation of phospholipase Cγ, as shown on primary endothelial cells [113].

An alternative calcium activation pathway was identified in the Rb-knockdown prostate cancer cell lines, in which kisspeptin 1 receptor was identified as a hypoxia response protein [114]. The action of kisspeptin on these cells differed between cell lines, with 22Rv1 cells exhibiting calcium mobilization, while the less-metastatic LNCaP cells showed none. The authors noted the potential use of the kisspeptin antagonists or agonists for treatment of metastatic, castration-resistant prostate cancer that these recent findings suggest. This pathway adds to the list of mechanisms that are likely to be impacted by cross-talk with intracellular calcium-mediated signaling involving molecules that range from the lipid-scavenging receptor CD36 and the extracellular calcium sensor CaSR to the multifunctional downstream factors such as myosin IC, as reviewed in Section 2.2 and Section 3.4 here and also recently [7].

Specific fatty acids appear to have a two-pronged effect on the regulation of angiogenesis. The HIF-1α induction and VEGF production pathway can be inhibited by *n*-3 polyunsaturated fatty acids (PUFA), as has been shown in human colon cancer cells [115]. The VEGF suppression mechanism is consistent with the results of the study that compared the Mediterranean (rich in ω-3 PUFA) and traditional Swedish diets [116]. The reduction of the serum *n*-6 to *n*-3 ratio in healthy subjects on the Mediterranean diet was found to be accompanied by a significant suppression of the levels of VEGF. Furthermore, it has been known for some time that ω-3 PUFA can lower the expression of VEGF receptor 2, as shown in experiments with docosapentaenoic acid on bovine aortic endothelial cells [117]. These in-vitro experiments demonstrated a functional effect on tube formation, whereas human umbilical vein cells showed reduced migration under the inhibitory action of conjugated eicosapentaenoic acid on the VEGF-induced vasculogenesis [118].

### 5.2. Involvement of Exosomes

A recent study [77,119] revealed a higher protein complement of exosomes secreted by cultured prostate cells (PC3 and LNCaP) subjected to hypoxia. In addition to being promising for biomarker development [120], these exosomes show direct mechanistic relevance to prostate cancer progression. Remarkably, they are smaller in size than those collected under the normoxia conditions, but are enriched in, among other proteins, heat shock proteins (HSP)-70 and -90, β-catenin, AKT, and IL6. Additionally, they exhibit an elevated metalloprotease activity and enhance the motility and invasivity of normoxic cells exposed to them. In normoxic PC3 cells treated with hypoxia-induced exosomes, elevated β-catenin levels were observed in the nucleus, and these cells exhibited a rise in the expression of E-cadherin.

The isoforms of HSP-70 and -90 that were detected at a higher level in the hypoxia-induced exosomes (type 8 and AB1, respectively) were distinct from those (type 5/GRP78 and B1/GRP94) known to be upregulated in the same cell lines subjected to a sustained calcium influx via a low dose of ionomycin [121]. However, an intriguing possible connection to the calcium regulation is suggested by the higher metalloprotease activity of hypoxia-induced exosomes. An enhanced HIF1-mediated expression and enzymatic activity of the metalloproteases in the hypoxia-treated cultured prostate cancer cells was reported previously [122]. The secretion of metalloprotease-containing exosomes, on the other hand, has most recently been found to be dependent in the PC3 cells on myosin IC [81], whose nucleocytoplasmic distribution is regulated by calcium [101].

A broader, paracrine-like effect of the hypoxia-induced exosomes has been linked in the cited study [77] to their elevated content of TGF-β2. This factor is known to promote differentiation of myofibroblasts and evolution of these cells’ cancer-promoting phenotype in mammary glands in an autocrine fashion [123]. The TGF-β2-containing, hypoxia-induced exosomes of epithelial prostate cancer cells had a similar effect on the prostate stromal cells in culture, manifested by an elevated expression of α-SMA, the marker of cancer-associated fibroblasts. These findings add to the growing list of exosome-mediated functions, which, as hypothesized above, may be under the kinetic control of the myosin-driven secretion subject to calcium and fatty acid regulation.

### 5.3. Related HIF-Dependent Mechanisms

A process related closely to the hypoxia response is reactive oxygen species (ROS)-mediated damage and the cellular responses to it. As demonstrated originally on pulmonary arterial smooth muscle cells [124], ROS stimulate HIF-1α expression via an induction of NF-κB. The latter factor effects the response by binding directly to an element that regulates the expression of HIF-1α. The sensitivity of this pathway to ROS has significance for radiation therapy. Supporting this notion, radiation has been found to induce HIF-1α expression in a number of tumor cell lines, including the prostate cancer DU145 cell line [125]. This effect can hypothetically be attributed to the reviewed action of ROS, which mediate much of the biological impact of radiotherapy [126]. Such an unintended downstream effect would contribute negatively to the treatment’s efficacy, which trails that of radical prostatectomy [127].

A similar mechanism of NF-κB mediated HIF-1 upregulation may be at play in prostate cancer cells, in light of the recent report that hypoxia in the androgen-independent cell lines leads to an upregulation of the bone metastasis-linked [128,129,130] chemokine receptor CX3CR1 in an HIF-1 and NF-κB-sensitive manner [131]. Knockdown of either factor was sufficient to suppress also the downstream CX3CR1-dependent effects that were observed, namely migration and invasion under hypoxic conditions in culture. Another remarkable downstream effect under these conditions is upregulation of GAPDH, which is also HIF1-dependent. These findings are in agreement with the observation that the transcriptional activity of HIF-1 correlates with tumorigenicity and metastatic potential among the prostate cancer cell lines [132]. In addition to low-oxygen zones of the primary tumor, as an environment that may suppress growth but induce an elevated metastatic potential, bone marrow is also a low-oxygen environment [133,134]. Secretion of cytokines by prostate cancer cells in culture under the action of hypoxia was found to enhance migration and vasculogenesis of bone marrow-derived endothelial progenitor cells [135]. This finding adds further complexity to the picture of prostate cancer cells’ metastatic phenotype induced by hypoxia via HIF-1α [136].

Cytosolic calcium-binding proteins S100A-8 and -9, whose expression is elevated in prostate adenocarcinoma tissues [137], were found to be upregulated in the BPH-1 prostate epithelial cell line in a hypoxia-induced manner via the HIF-1α binding elements in their promoters [138]. The downstream effects of these calcium-binding proteins, which have been demonstrated in other biological contexts, include activation of Toll-like receptors [139], as well as induction of a pro-inflammatory environment and carcinogenesis [140]. Further underscoring the linkage to reviewed mechanisms of the hypoxia response, expression of S100A-8 and -9 in stromal cells of premetastatic lungs has been found to be induced by VEGF (in addition to TGF-β and TNFα) that is secreted by the primary tumor [141], while in hepatocellular carcinoma cells their expression is promoted by NF-κB [142]. It will be important to investigate if VEGF-mediated feedback also exists in the cancerous prostate, and what effect modulation by calcium-binding has in prostate cancer on the reviewed NF-κB-mediated induction of HIF-1 itself.

### 5.4. Connection to Castration Resistance

In light of the importance of AR overexpression for the emergence of castration resistance, as demonstrated in particular by its necessary and sufficient role in the xenograft models [143], another aspect of HIF-1 function and regulation acquires a special significance. Androgen ablation (both surgical and chemical) induces hypoxia in the prostate, at least initially [144,145]. This is accompanied by an upregulation of HIF-1 [144,146]. HIF-1, in turn, increases expression of AR and downstream AR-dependent transcription, such as that of *NXK3.1* [147] and PSA [148], even in the very low androgen concentration associated with castration—0.1 nM of 5α-DHT [147].

Of further relevance to the mechanism of castration resistance emergence and to the identification of predictive markers for this lethal transformation is the recent research into HIF-1α expression under normoxia conditions [149]. In this work, HIF-1α expression was found to be correlated with androgen sensitivity among prostate cancer cell lines, and the difference was attributable to the factor’s translation efficiency. Moreover, a HIF-1α protein knockdown reduced the cells’ viability and migration. At the same time, Kaplan-Meier analysis showed the decreased metastasis-free and castration resistance-free survival of those patients on androgen deprivation therapy whose tumors expressed HIF-1α. On the one hand, these findings suggest a use for the HIF-1α expression as a prognostic marker for poor response to the first-line therapy. On the other hand, they point to a special significance of mechanisms other than hypoxia that can result in the factor’s activation, such as its calcium regulation. At the same time, specifically nuclear localization of HIF-1α was found to be a marker for good prognosis (alongside low EGFR) among patients receiving radiation therapy, with or without a concomitant androgen deprivation [150]. The latter finding, while it may reflect a difference brought about by the radiation therapy, also calls attention to the emerging theme of nucleocytoplasmic partitioning as a likely reciprocal modulator of nuclear (e.g., transcription) and cytoplasmic (e.g., migration) functions of the multifunctional proteins in prostate cancer (see also [7]).

Meanwhile, based on literature meta-analysis [151], it had been proposed that HIF-1α could be targeted to improve the response to conventional treatment modalities, including chemotherapy and radiotherapy, in addition to androgen deprivation. Research into the metabolic regulation mediated by this factor, however, suggested that better druggable targets may be found among the enzymes and transporters lying downstream from HIF-1 [152]. At the same time, a retrospective analysis of metastasis-free and castration resistance-free survival of patients on non-specific inhibitors of HIF-1 (digoxin, metformin, and angiotensin-2 receptor blockers) showed a significant positive effect [153]. Collectively, the data suggest a central role of the calcium- and fatty acid-regulated HIF functions that connect multiple pathways involved in prostate cancer progression and therapy response. Although more work is needed in this area, the dependence may be indicative of the mechanisms behind the diet-linked epidemiological effects of these nutrients’ co-consumption.

## 6. Metastasis and Bone Tropism

### 6.1. Connection with Obesity and Fatty Acid Transfer

The evidence for fatty acids’ involvement in the progression of prostate cancer to metastasis and bone tropism to date comes from general epidemiological studies, work addressing the tissue cell types’ interaction, and in-vitro experiments. However, the picture is far from complete. Obesity has been found to be associated with negative prognosis and, specifically, with progression to aggressive metastatic disease [154,155,156,157]. Tissue culture experiments suggest that the association may be linked to the adipocyte-prostate epithelial cell interaction. In three-dimensional co-culture, prostate cancer cell lines exhibited enhanced growth, dedifferentiation, and signs of EMT [158]. In a study with deuterated palmitic acid [159], it was established that larger adipocytes accumulate a higher proportion of this fatty acid and are able to transfer it to the bone-metastatic prostate cancer cells in the co-culture. This finding is suggestive in the context of the association with adipocytes in lipid-rich areas of the bone marrow and fatty acid uptake, both of which are known to be exhibited by the bone-metastatic cells [160]. Moreover, the same study showed that invasion of prostate cancer cells and their migration toward bone marrow stroma are promoted by the ω-6 PUFA, arachidonic acid. This effect is mediated by the arachidonic acid’s conversion into, in particular, prostaglandin E2 (PE2), and is reversed by the ω-3 PUFA (eicosapentaenoic, and docosapentaenoic acids), apparently through a competitive inhibition mechanism. Such an action would be similar to the ω-3 sensitivity of the earlier-reported proliferative and anti-apoptotic action of arachidonic acid [161,162,163].

It should be noted, in particular, that the anti-apoptotic effect of arachidonic acid on prostate cancer cells [163,164] is connected with the activity of 5-lipoxygenase and production of 5(*S*)-hydroxyeicosatetraenoic acid (5(*S*)-HETE) and 5-oxo-eicosatetraenoic acid (5-oxo-ETE). These effects were consistent with the earlier finding of the 5-lipoxygenase-dependent promotion of prostate cancer cells’ proliferation by arachidonic acid [165]. The differential expression of 5-oxo-ETE receptor in prostate cancer cells [166] enables transmission of the signal to these downstream effects through phospholipase Cβ and diacylglycerol and protein kinase Cε [167,168]. Interestingly, the most recent work shows that testosterone can bind the same receptor and antagonize some the effects of 5-oxo-ETE [169]. Although the above-described growth and anti-apoptotic effects were not mediated by the products of 12-lipoxygenase, the latter pathway also plays a variety of roles in prostate cancer, including the 12(*S*)-HETE mediated secretion of MMP9 and VEGF [170,171,172]. Of special relevance to the acquisition of the distant-metastatic phenotype by prostate cancer cells is the effect of 12(*S*)-HETE whereby it suppresses [173] the detachment-induced apoptosis (anoikis, whose mechanisms will be further reviewed in Section 6.3).

At the same time, the mechanistic details of the fatty acid-mediated interaction with the stroma are beginning to emerge from studies that have focused on the lipid chaperone, fatty acid binding protein 4 (FABP4). Bone marrow adipocytes can supply fatty acids to the metastatic prostate cells and induce their invasiveness under conditions of high-fat diet through a mechanism that involves upregulation of FABP4 in the prostate-derived cells [155,174]. The most recent work [175] positioned FABP4 at the crossroads of the epithelial-stroma interaction by demonstrating that its secretion leads to MMP upregulation and interleukin production in the stroma cells. The FABP4 plasma level, at the same time, was found to be associated with the aggressive prostate cancer phenotype. The fatty acid-mediated and diet-modulated tumor stroma interaction can be expected to remain an active area of research. One additional question raised by the reviewed work is the likely importance of the secretion kinetics in the metastatic prostate cancer cells for the establishment of bone tropism.

### 6.2. Calcium Sensitivity

Apart from the intracellular calcium homeostasis, prostate cancer metastasis has an intriguing bidirectional relationship with extracellular calcium. Hypocalcemia due to the uptake by osteoblastic metastases feeds back on the metastatic process through expression of the parathyroid hormone receptor [176]. The intracellular signaling induced by the elevated calcium ion concentration in the bone environment similarly promotes bone-tropic metastasis. The seminal work done on prostate cancer cell lines in vitro and in vivo demonstrated that proliferation of skeletal metastatic cells, unlike non-skeletal ones, is promoted by the higher extracellular calcium concentration characteristic of the bone microenvironment [177]. The calcium acts on the G protein-coupled receptor CaSR, which inhibits intracellular cAMP accumulation, counteracting, in particular, the signaling induced by forskolin and PE2. Knockdown of this receptor is sufficient to inhibit metastatic progression in vivo. Among the downstream signaling molecules, as the same study shows, the AKT pathway is involved in the enhancement of the attachment of PC3 cells under the high-calcium conditions. Underscoring the significance of these findings to the mechanisms of human disease, the CaSR expression, as determined by immunohistochemistry of patient samples, is correlated with lethal progression [178].

It should be mentioned in this connection that cyclin D1, which is normally synthesized and imported into the nucleus during interphase in preparation for DNA replication [179], was found to be sensitive to extracellular calcium concentration in skeletal metastatic PC3 cells: Its degradation under the conditions of serum starvation was abolished when the extracellular concentration mimicked that of the bone microenvironment. These results indicate the existence of a critical junction between androgen deprivation and the skeletal metastasis in prostate cancer that involves the calcium-regulated nuclear import. It appears likely that the cyclin D1-mediated effect may be regulated downstream of CaSR.

CaSR has varying roles in tumorigenesis in different organs [180]. A number of single-nucleotide polymorphisms in CaSR have been identified that exhibit an association with prostate cancer recurrence [181] and lethality [182]. The former effect is dependent on the dietary calcium intake. In agreement with the cited work of Liao et al. [177], bone-metastatic prostate cancer is characterized by a higher expression of CaSR [183], as graded either by a pathologist or algorithmically. Although prostate cancer metastasis to the bone is predominantly osteoblastic, and the humoral hypercalcemia of malignancy syndrome in the patients is atypical, the canonical activation of parathyroid hormone-related peptide (PTHrP) production by CaSR, which is characteristic of osteoclastic metastasis—for example, in breast and lung cancer—still appears to be relevant [180]. Polycationic agonists of CaSR induce PTHrP secretion in PC3 and LNCaP cell lines, both of which express the receptor [184]. Evidence has been obtained that points to a transactivation of EGFR via a membrane metalloprotease and autocrine shedding of heparin-binding EGF behind this induction [185]. Collectively, the CaSR-mediated effects on prostate cancer metastasis to the bone provide a mechanistic framework in which to study the potential prognostic markers and therapeutic targets that may be associated with the reviewed epidemiologic dependence on calcium intake.

### 6.3. PTHrP Signaling

The bone-tropic PC3 cells respond in culture to the N-terminal PTHrP fragment with an increased proliferation, whereas in the LNCaP cells, whose growth is androgen-sensitive, this reaction is androgen-dependent [186]. While the PTH domain used in this study excluded the peptide’s NLS, it is possible that the full-length PTHrP acts in an intracrine manner. The importin-β1-mediated nuclear import of endogenous PTHrP has been demonstrated in a number of other cell types [187]. The nuclear and nucleolar accumulation of this peptide can also be induced by the nuclear export inhibitor leptomycin B (ibid.), suggesting that its regulation is via a specific balance in the bidirectional transport.

Recent xenograft and in-vitro experiments [188] indicate that progression and metastasis are controlled by the level of expression of PTHrP in epithelial prostate cancer cells. This is correlated with the expression of EMT markers, including a downregulation of E-cadherin, and in vivo, with a sharp increase of bone-resorption capacity. A transformation related to EMT is resistance to anoikis, the detachment-induced apoptosis that can be induced during bloodstream and lymphatic-system dissemination of the cancer cells [189]. The anoikis of prostate cancer cells in vitro was found to be negatively regulated by PTHrP via an intracrine mechanism dependent on the peptide’s NLS [190]. A gene array analysis showed that the effect is mediated by suppression of the mRNA-level expression of proapoptotic factor TNFα. Knockdown of PTHrP reduced skeletal metastasis in a mouse xenograft model, a further indication that the intracrine, nuclear PTHrP protects cells suspended in the bloodstream in the course of the distant-site metastasis. At the same time, there is evidence that intracrine PTHrP nuclear import may also be effected through a pathway independent of its canonical NLS. Acting in this manner, the peptide may be capable of inducing expression of IL8, whereas expression of VEGF in these experiments was dependent on the PTH/PTHrP receptor-binding domain [191]. Expression of both IL8 and VEGF is characteristic of carcinoma of the prostate, as contrasted with benign prostatic hyperplasia and normal tissue [192]. IL8 is not only capable of stimulating growth, MMP9 secretion, and invasivity of the epithelial prostate cancer cells in vitro, but also of promoting metastasis marked by MMP9 production and enhanced neovascularity [193]. Thus, the downstream effects of the calcium-induced PTHrP signaling may be mediated by partially redundant pathways that involve paracrine and autocrine membrane receptor activation, as well as by an intracrine action through the canonical and noncanonical nuclear import mechanisms. The likely multilayered redundancy behind the pathophysiological effects of this calcium-induced signaling modality merits attention in the pathway-based design of future therapeutic countermeasures to prostate cancer metastasis.

**Figure 2:**
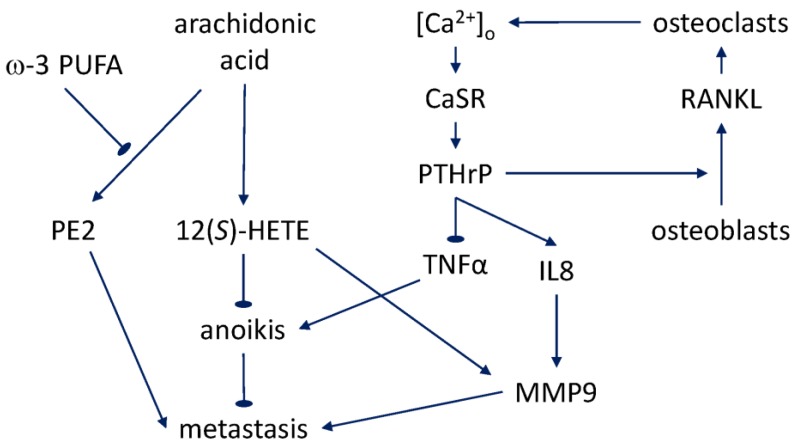
Some of the pathways involved in the fatty acid and calcium regulation of prostate cancer metastasis.

The skeletal complications during protracted bone metastasis, including pain and fractures, significantly impact the quality of life of prostate cancer patients [194]. At the tissue and molecular levels, recruitment of osteoblasts and osteoclasts, bone remodeling, and liberation of growth factors from the bone matrix feeds back on metastatic proliferation in what has been referred to as a “vicious cycle” [195]. PTHrP produced by metastatic prostate cancer cells stimulates receptor activator of nuclear factor κB ligand (RANKL) production by osteoblasts [196]. RANKL, in turn, acts on osteoclasts, which express the receptor (RANK) for the ligand. Furthermore, the calcium-regulated PTHrP secretion turns out to be a mechanism behind the formation of osteoclastic lesions in prostate cancer [197]. Release and activation of the matrix-stored factors, including TGF-β, promote further growth of the metastatic mass [198]. In addition to being able to regulate the cell types of the bone microenvironment that express RANK, metastatic prostate cells themselves express this receptor and therefore also respond directly to RANKL with enhanced growth and invasive motility [199,200]. This regulation is effected via the ERK1/2 and JNK pathways. The expression of PTHrP and RANKL in metastatic prostate cancer cells is controlled by NF-κB and leads to development of the characteristic pattern of osteoblastic and osteoclastic mixed tumor [201]. In sum, the varied targets of the PTHrP signaling appear to close feedback loops involved in prostate cancer metastasis to the bone (Figure 2). The quantitative interplay of these mechanisms and the dietary calcium regulation will likely prove fruitful as directions for future research.

## 7. Summary and Outlook

Epidemiological, nutritional, and molecular-biological research continues to reveal the multifaceted aspects of the regulation of prostate cancer progression by the ubiquitous and linked factors of the fatty acid and calcium intake. In light of the most recent work, both extracellular calcium and fatty acid availability influence intracellular signaling via specific receptors. Both signals can be converted into intracellular calcium transients, although the details of this mechanism’s implementation in epithelial prostate cancer cells remains to be investigated. Recent research demonstrates that the critical steps toward the unfavorable outcome in prostate cancer—castration resistance and metastasis—are regulated by various intracellular-calcium orchestrated signaling pathways, and also directly by extracellular calcium and fatty acids.

Open questions concern the regulatory processes impacting the progression to malignancy following escape from under the control of the anti-androgen therapy, the complex set of responses to hypoxia, and bone tropism. The perturbation of fatty acid uptake and metabolism during the transition to hormone independence is likely to alter the composition of the membranes of secretory vesicles, including exosomes, and thereby modulate the kinetics of secretion driven by the lipid interaction of molecular motors. According to the most recent work, such a mechanism may represent a step not only towards the modulation of the secretion of the enzymes responsible for the invasive properties of the primary tumor, but also towards the exosome-mediated intercellular communication leading to the propagation of the malignant phenotype among the cellular subpopulations in the tumor mass. On the whole, the picture currently emerging from the fatty acid and calcium regulation studies is indicative of the rising recognition of the possibly pivotal role of these mechanisms in prostate cancer progression. It may be expected, therefore, that the research currently underway in this field will be able to identify additional prognostic markers and key targets for differential diagnosis of the malignant forms and, subsequently, effective therapeutic intervention.

## Abbreviations

AR	Androgen receptor
CAMK	Calcium-calmodulin dependent kinase
CaSR	Calcium-sensing receptor
DHT	Dihydrotestosterone
EGF	Epidermal growth factor
EGFR	EGF receptor
EMT	Epithelial-mesenchymal transition
ERK	Extracellular signal-regulated kinase
ETE	Eicosatetraenoic (acid)
FABP	Fatty acid-binding protein
FAS	Fatty acid synthase
HETE	Hydroxyeicosatetraenoic (acid)
HIF	Hypoxia-induced (transcription) factor
HSP	Heat shock protein
LDL	Low density lipoprotein
LDLR	LDL receptor
MMP	Matrix metalloproteases
NLS	Nuclear localization signal (sequence)
PE2	Prostaglandin E2
PSA	Prostate-specific antigen
PTHrP	Parathyroid hormone-related peptide
PUFA	Polyunsaturated fatty acid
RANK	Receptor activator of nuclear factor κB
RANKL	RANK ligand
ROS	Reactive oxygen species
SOCE	Store-operated calcium entry
TGF	Transforming growth factor
TRAMP	Transgenic adenocarcinoma of the mouse prostate
VEGF	Vascular endothelial growth factor
